# Selecting β-glucosidases to support cellulases in cellulose saccharification

**DOI:** 10.1186/1754-6834-6-105

**Published:** 2013-07-24

**Authors:** Hele Teugjas, Priit Väljamäe

**Affiliations:** 1 Institute of Molecular and Cell Biology, University of Tartu, Riia 23b – 202, 51010, Tartu, Estonia

**Keywords:** Cellulase, Cellulose, β-glucosidase, Cellobiose, Glucose, Inhibition, *Acremonium thermophilum*, *Thermoascus aurantiacus*

## Abstract

**Background:**

Enzyme end-product inhibition is a major challenge in the hydrolysis of lignocellulose at a high dry matter consistency. β-glucosidases (BGs) hydrolyze cellobiose into two molecules of glucose, thereby relieving the product inhibition of cellobiohydrolases (CBHs). However, BG inhibition by glucose will eventually lead to the accumulation of cellobiose and the inhibition of CBHs. Therefore, the kinetic properties of candidate BGs must meet the requirements determined by both the kinetic properties of CBHs and the set-up of the hydrolysis process.

**Results:**

The kinetics of cellobiose hydrolysis and glucose inhibition of thermostable BGs from *Acremonium thermophilum* (*At*BG3) and *Thermoascus aurantiacus* (*Ta*BG3) was studied and compared to *Aspergillus sp.* BG purified from Novozyme®188 (*N188*BG). The most efficient cellobiose hydrolysis was achieved with *Ta*BG3, followed by *At*BG3 and *N188*BG, whereas the enzyme most sensitive to glucose inhibition was *At*BG3, followed by *Ta*BG3 and *N188*BG. The use of higher temperatures had an advantage in both increasing the catalytic efficiency and relieving the product inhibition of the enzymes. Our data, together with data from a literature survey, revealed a trade-off between the strength of glucose inhibition and the affinity for cellobiose; therefore, glucose-tolerant BGs tend to have low specificity constants for cellobiose hydrolysis. However, although a high specificity constant is always an advantage, in separate hydrolysis and fermentation, the priority may be given to a higher tolerance to glucose inhibition.

**Conclusions:**

The specificity constant for cellobiose hydrolysis and the inhibition constant for glucose are the most important kinetic parameters in selecting BGs to support cellulases in cellulose hydrolysis.

## Background

Cellulose is the most abundant biopolymer on Earth and has a great potential as a renewable energy source. The enzymatic hydrolysis of cellulose, followed by fermentation to ethanol is a promising green alternative for the production of transportation fuels. In nature, cellulose is degraded mostly by fungi and bacteria, which secret a number of hydrolytic and oxidative enzymes [1,2], though fungal enzymes have received most of the attention to date regarding biotechnological applications. The major components of fungal cellulase systems are cellobiohydrolases (CBHs), exo-acting enzymes that processively release consecutive cellobiose units from cellulose chain ends. Endoglucanases (EGs) attack cellulose chains at random positions and work in synergism with CBHs. The hydrolysis of cellulose is completed by β-glucosidases (BGs), which hydrolyze cellobiose and soluble cellodextrins to glucose [3]. BGs can be found in glycoside hydrolase (GH) families 1, 3, 9, 30 and 116 [4,5], and most of the microbial BGs employed in cellulose hydrolysis belong to GH family 3 [6]. Because cellobiose is a strong inhibitor of CBHs, the BG activity in cellulase mixtures must be optimized to overcome the product inhibition of CBHs. The inhibition of BGs by glucose must also be considered because the accumulation of glucose will lead to the accumulation of cellobiose and CBH inhibition. Many BGs are also inhibited by their substrate, and this apparent substrate inhibition is caused by the transglycosylation reaction, which competes with hydrolysis [7,8]. The catalytic mechanism of retaining BGs involves a covalent glucosyl-enzyme intermediate [9], which may be attacked by water (hydrolysis) or by a hydroxyl group of the substrate (transglycosylation). In addition to the substrate, attack by other nucleophiles, such as alcohols, can also lead to transglycosylation [9]. Transglycosylation is under kinetic control, meaning that all cellobiose and transglycosylation products will eventually be hydrolyzed to glucose.

To be economically feasible, the hydrolysis of cellulose must be conducted at a high dry matter concentration, which inevitably results in a high concentration of hydrolysis products, cellobiose and glucose, and makes the product inhibition of enzymes a major challenge in process and enzyme engineering. Several process set-ups have been developed that minimize product inhibition, and bioreactors enabling the continuous removal of hydrolysis products have been constructed [10,11]. The most often applied set-up is simultaneous saccharification and fermentation (SSF), whereby glucose is constitutively removed by fermentation to ethanol. To bypass the use of BGs, yeast strains capable of fermenting cellobiose and cellodextrins have also been generated [12]. A major drawback of SSF is with regard to the different optimal conditions for the enzymatic hydrolysis of cellulose and yeast fermentation. The optimal temperature for yeast is 35°C, whereas cellulases exhibit the highest activity at temperatures of 50°C or higher. Although both processes can be conducted at each optimal temperature in separate hydrolysis and fermentation (SHF), the enzymes must operate under conditions of severe product inhibition [13]. An alternative process in between conventional SHF and SSF employs the high-temperature partial pre-hydrolysis of cellulose, followed by SSF [14]. Thus, the properties of candidate enzymes, such as temperature optima and tolerance toward inhibitors, must be selected depending on the process set-up.

In this study, we characterize the thermophilic GH family 3 BGs from *Acremonium thermophilum* (*At*BG3) and *Thermoascus aurantiacus* (*Ta*BG3) [15,16] in terms of cellobiose hydrolysis and glucose inhibition; a well-characterized BG from *Aspergillus sp*, purified from Novozyme®188 (*N188*BG), was used for comparison. A literature survey was also performed to identify correlations between the kinetic parameters of cellobiose hydrolysis and glucose inhibition.

## Results and discussion

### Kinetics of cellobiose hydrolysis

The hydrolysis of cellobiose by BGs, *At*BG3, *Ta*BG3 and *N188*BG was monitored by measuring the initial rates of glucose formation (*v*_Glc_). The values of the observed rate constants for cellobiose turnover (*k*^obs^_CB_) were derived from *v*_Glc_ and the total concentration of enzyme ([E]_0_) according to

(1) $$k_{\mathrm{CB}}^{\mathrm{obs}}=\frac{1}{2}\frac{v_{\mathrm{Glc}}}{{\left[E\right]}_{0}}$$

**Figure 1:**
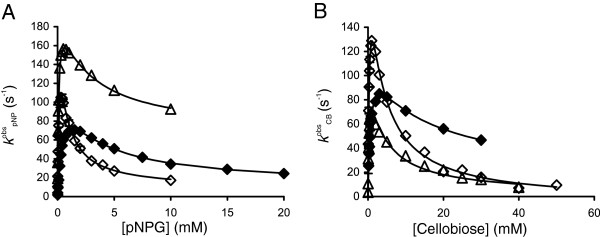
**Hydrolysis of pNPG and cellobiose by β-glucosidases.** Observed rate constants (*k*^obs^) for the β-glucosidase-catalyzed turnover of pNPG **(panel A)** and cellobiose **(panel B)** at 25°C. β-glucosidases included *Ta*BG3 (◊), *At*BG3 (∆) or *N188*BG (♦). The solid lines are from the non-linear regression according to Equation 2.

**Scheme 1:**
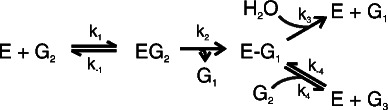
**Schematic representation of the β-glucosidase-catalyzed turnover of cellobiose.** Cellobiose (G_2_) binds to the enzyme to form a Michaelis complex (EG_2_) that reacts to form a first product (glucose, G_1_) and a covalent glucosyl-enzyme intermediate (E-G_1_). The latter can react with water to produce glucose (hydrolysis) or with cellobiose to produce a trisaccharide, G_3_, as a second product (transglycosylation). In the case of such model substrates as pNPG or MUG, the chromophore is released as the first product.

The hydrolysis kinetics of a chromogenic model substrate, para-nitrophenyl-β-glucoside (pNPG), was also studied. In this case, the initial rates of the liberation of para-nitrophenol (pNP) (*v*_pNP_) were monitored, and the observed rate constants for pNPG turnover (*k*^obs^_pNPG_) were calculated as *v*_pNP_/[E]_0_. All BGs were found to subjected to substrate inhibition using both pNPG and cellobiose as substrates (Figure 1). The substrate inhibition of BGs is a well-known phenomenon that is caused by the competition between water (hydrolysis) and substrate (transglycosylation) for the glucosyl-enzyme intermediate (Scheme 1) [7,8]. The dependency of *k*^obs^_CB_ (and also *k*^obs^_pNPG_) on the substrate concentration is given by a set of four parameters, catalytic constants *k*_cat(h)_ and *k*_cat(t)_ and Michaelis constants *K*_M(h)_ and *K*_M(t)_ for hydrolysis and transglycosylation, respectively [17,18].

(2) $$k_{\mathrm{CB}}^{\mathrm{obs}}=\frac{k_{\mathrm{cat}\left(h\right)}K_{M\left(t\right)}\left[\mathrm{CB}\right]+\frac{1}{2}k_{\mathrm{cat}\left(t\right)}{\left[\mathrm{CB}\right]}^{2}}{K_{M\left(t\right)}K_{M\left(h\right)}+K_{M\left(t\right)}\left[\mathrm{CB}\right]+{\left[\mathrm{CB}\right]}^{2}}$$

**Figure 2:**
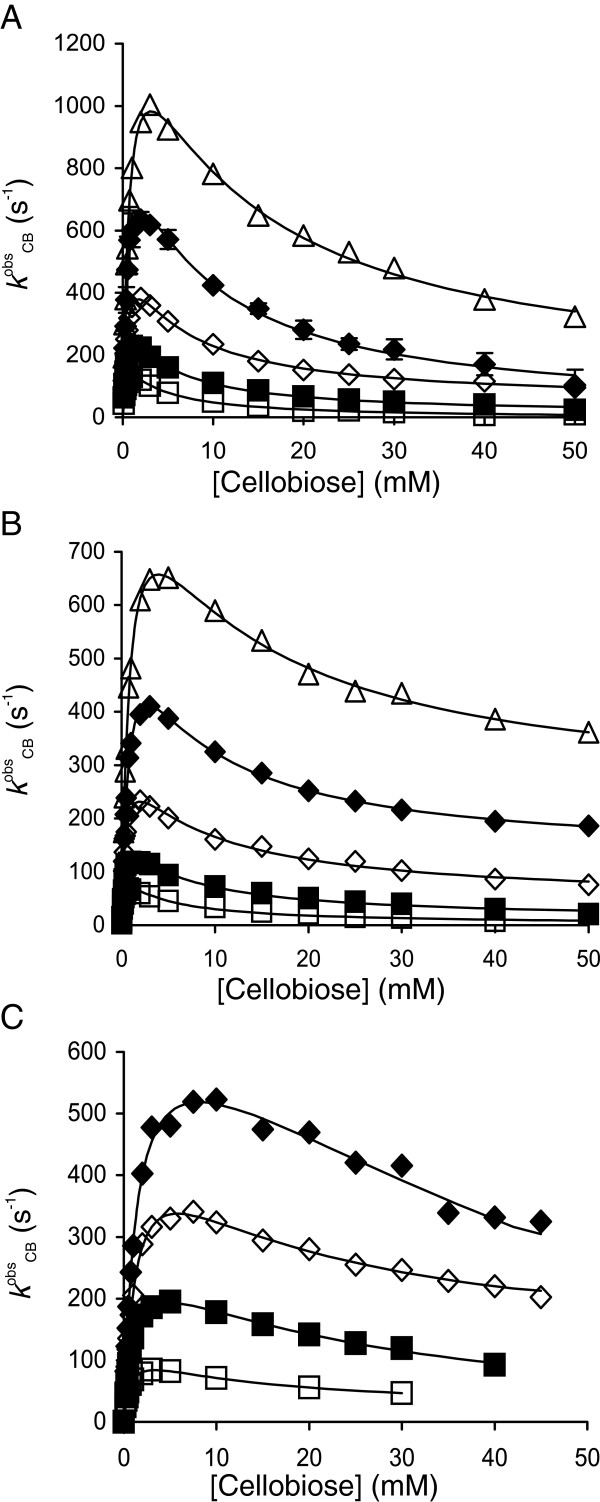
**Hydrolysis of cellobiose at different temperatures.** Observed rate constants for the turnover of cellobiose (*k*^obs^_CB_) at 25°C (□), 35°C (■), 45°C (◊), 55°C (♦) and 65°C (∆). β-glucosidases included **(A)***Ta*BG3, **(B)***At*BG3 and **(C)***N188*BG. The solid lines are from the non-linear regression according to Equation 2.

All four parameters in Equation 2 are combinations of the rate constants in Scheme 1[7,8]. The hydrolysis of cellobiose results in the formation of two molecules of glucose, whereas transglycosylation results in the formation of one molecule of glucose and one trisaccharide (Scheme 1). For this reason, the catalytic constant for transglycosylation in Equation 2 is multiplied by a factor of ½; this correction is not necessary for the pNPG substrate, as both the hydrolysis and transglycosylation reactions result in the formation of one molecule of pNP. The values of all four parameters were found by the non-linear regression analysis of the data for cellobiose turnover, according to Equation 2. We were primarily interested in the hydrolytic reaction. Therefore, the data points in the region of high cellobiose concentrations were, in some cases, insufficient for precise measurements of the parameter values for transglycosylation. However, one can estimate that the values of *k*_cat(h)_ were approximately an order of magnitude higher than the values of *k*_cat(t)_, whereas the opposite was true for the *K*_M_ values (Additional file 1: Table S1). To test the possible interdependency between the parameters for the hydrolytic and transglycosylation reactions, we performed a non-linear regression analysis with the datasets in which the highest cellobiose concentration was limited to 5 *K*_M(h)_ (Additional file 1: Table S1). The resulting *k*_cat(h)_ and *K*_M(h)_ values were close to those obtained from the analysis of the full datasets, indicating that the values for *k*_cat(h)_ and *K*_M(h)_ can be calculated without precise estimates of the values of *k*_cat(t)_ and *K*_M(t)_ (Additional file 1: Table S1). Another possibility for determining the values of *k*_cat(h)_ and *K*_M(h)_ is to restrict the analysis to data points in the regions of substrate concentration at which substrate inhibition is not yet revealed and to employ the simple Michaelis-Menten equation. However, this approach resulted in somewhat lower *k*_cat(h)_ and *K*_M(h)_ values, whereas the values of *k*_cat(h)_/*K*_M(h)_ were overestimated (Additional file 1: Table S1). Figure 2 shows the turnover of cellobiose at different temperatures, and the *k*_cat(h)_ and *K*_M(h)_ values obtained are listed in Table 1. Although at the same order of magnitude, the highest *k*_cat(h)_ values were found for *Ta*BG3, followed by *N188*BG and *At*BG3. However, it must be noted that, because of the competing transglycosylation reaction, cellobiose hydrolysis at the *k*_cat(h)_ value is never realized (*k*_cat(h)_ is the limiting value of *k*^obs^_CB_ in the absence of transglycosylation, see Equation 2 in the case of *k*_cat(t)_ = 0 and *K*_M(t)_ → ∞). The highest measured *k*^obs^_CB_ values averaged 60% ± 4%, 81% ± 13% and 72% ± 3% percent of the *k*_cat(h)_ value for *Ta*BG3, *At*BG3 and *N188*BG, respectively (Table 1). The highest *k*_cat(h)_/*K*_M(h)_ values were found for *Ta*BG3, followed by *At*BG3 and *N188*BG (Table 2). The values of all the kinetic parameters increased with increasing temperature. The activation energies for *k*_cat(h)_ and *k*_cat(h)_/*K*_M(h)_ and standard enthalpy changes for *K*_M(h)_ and *K*_i_ were derived from the corresponding Arrhenius plots (Additional file 1: Figure S1) and are listed in Table 3. Among the parameters examined, the highest activation energies were found for *k*_cat(h)_; activation energies for cellobiose hydrolysis in the range of 50 kJ mol^-1^ have previously been reported for BGs, consistent with our observations [19].

**Table 1:** Kinetic parameters for cellobiose hydrolysis by β-glucosidases

	***k***_**cat(h) **_**(s**^**-1**^)^**a**^			***K***_**M(h)**_**(mM)**^**a**^		
**t (°C)**	***N188*BG**	***Ta*BG3**	***At*BG3**	***N188*BG**	***Ta*BG3**	***At*BG3**
25	121 ± 4 (70%)	227 ± 10 (57%)	105 ± 11 (95%)	0.73 ± 0.04	0.42 ± 0.03	0.29 ± 0.07
35	271 ± 5 (72%)	401 ± 19 (57%)	180 ± 11 (92%)	0.97 ± 0.04	0.51 ± 0.04	0.32 ± 0.04
45	493 ± 12 (70%)	632 ± 20 (61%)	326 ± 14 (81%)	1.34 ± 0.06	0.60 ± 0.03	0.41 ± 0.04
55	691 ± 27 (76%)	1058 ± 81 (60%)	666 ± 23 (66%)	1.36 ± 0.12	0.67 ± 0.10	0.87 ± 0.05
65		1497 ± 53 (67%)	968 ± 31 (71%)		0.82 ± 0.06	0.93 ± 0.06

The values in parentheses show the highest measured value of the rate constant for cellobiose hydrolysis as a percentage of *k*_cat(h)_.

^a^The values of *k*_cat(h)_ and *K*_M(h)_ were determined by a non-linear regression analysis of the data of cellobiose turnover, according to Equation 2.

**Table 2:** Specificity constants of β-glucosidases for cellobiose

	***k***_**cat(h)**_/***K***_**M(h)**_**for cellobiose (x 10**^**5**^ **M**^**-1**^ **s**^**-1**^**)**^**a**^		
**t (°C)**	***N188*BG**	***Ta*BG3**	***At*BG3**
25	1.66 ± 0.10	5.43 ± 0.45	3.61 ± 0.84
35	2.78 ± 0.11	7.81 ± 0.67	5.62 ± 0.77
45	3.69 ± 0.17	10.6 ± 0.60	7.99 ± 0.77
55	5.07 ± 0.44	15.5 ± 2.16	7.65 ± 0.47
65		18.4 ± 1.25	10.4 ± 0.63

^a^The *k*_cat(h)_/*K*_M(h)_ values were calculated from the values of *k*_cat(h)_ and *K*_M(h)_ listed in Table 1.

**Table 3:** Activation energies and binding enthalpies for the kinetic parameters of β-glucosidases

	**Activation energy, *E***_**a **_**(kJ mol**^**-1**^**)**^**a**^		**Binding enthalpy, Δ*H***^**0 **^**(kJ mol**^**-1**^**)**^**a**^	
	***k***_**cat(h)**_	***k***_**cat(h)**_/***K***_**M(h)**_	***K***_**M(h)**_	***K***_**i(Glc)**_
*N188*BG	47.6 ± 1.3	29.5 ± 1.7	18.1 ± 1.0	19.6
*Ta*BG3	39.8 ± 1.9	26.2 ± 2.3	13.6 ± 1.2	22.8
*At*BG3	48.2 ± 2.7	20.5 ± 2.4	27.7 ± 3.3	24.6

^a^For the parameter p, the activation energy (for *k*_cat(h)_ and *k*_cat(h)_/*K*_M(h)_) and standard binding enthalpy (for *K*_M(h)_ and *K*_i(Glc)_) was obtained from the slope of the line in the coordinates of ln(p) *versus* 1/T. For the data, see Additional file 1: Figure S1.

### Inhibition of β-glucosidases by glucose

Glucose inhibition was evaluated using pNPG or 4-methylumbelliferyl-β-glucoside (MUG) as the substrate. The dependency of the strength of glucose inhibition on the substrate used for inhibition studies reported in the literature, i.e., chromogenic model substrates or cellobiose, is controversial. In some studies, glucose inhibition appears stronger with a cellobiose than pNPG substrate [20], whereas the opposite is also reported [20-24]. Furthermore, there is no obvious mechanistic interpretation for why the inhibition strength should be different with cellobiose and pNPG or MUG. In all cases, nucleophilic attack results in the formation of the same glucosyl-enzyme intermediate [9], and the only difference lies in the nature of the leaving group in the +1 binding site, which is glucose in the case of cellobiose and para-nitrophenole (pNP) or 4-methylumbelliferone (MU) in the case of pNPG or MUG, respectively. Therefore, we chose to study glucose inhibition on model substrates, the hydrolysis of which can be easily detected in a background of added glucose.

**Figure 3:**
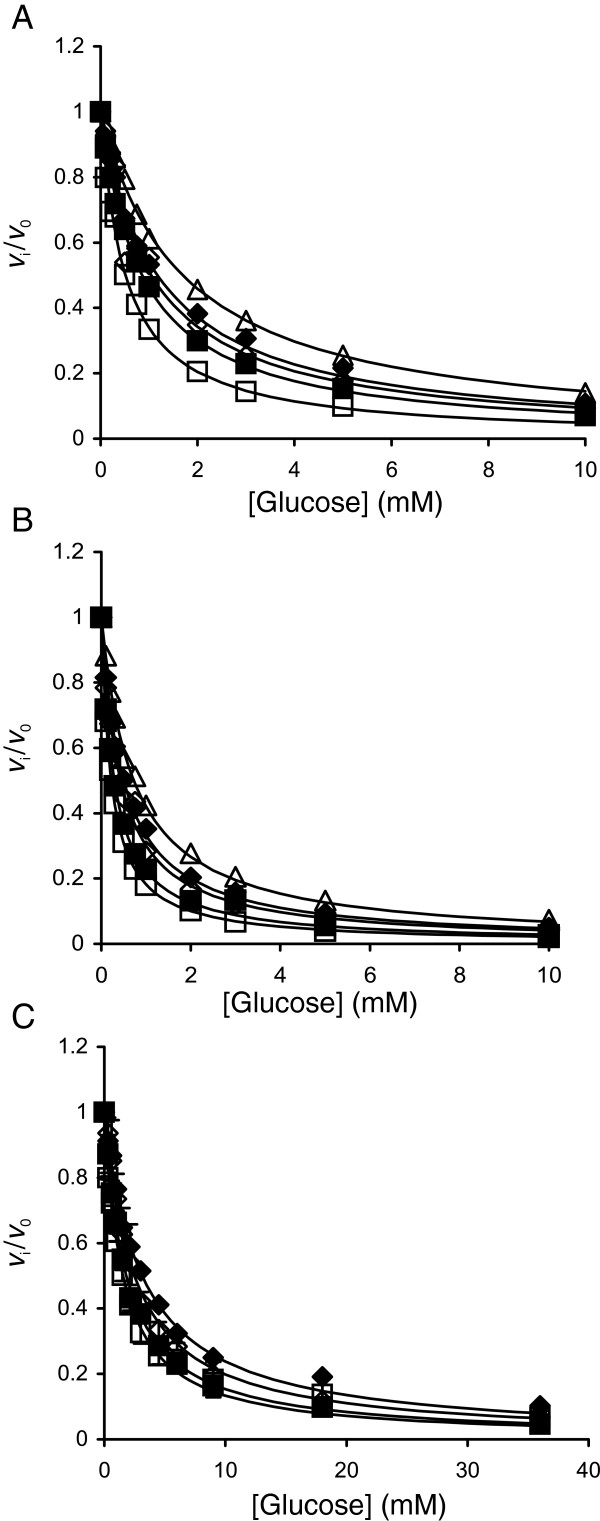
**Glucose inhibition of β-glucosidases.** The initial rates of the hydrolysis of 5 μM MUG by *Ta*BG3 **(A)**, 2.5 μM MUG by *At*BG3 **(B)** or 50 μM pNPG by *N188*BG **(C)** measured in the presence of glucose (*v*_i_) were divided by those measured in the absence of glucose (*v*_0_). The temperatures used were 25°C (□), 35°C (■), 45°C (◊), 55°C (♦) and 65°C (∆). The solid lines are from the non-linear regression according to Equation 3.

Although not without exceptions [25], glucose is a competitive inhibitor for BGs. In one trial (25°C, pNPG) we tested the type of inhibition by assessing the influence of glucose on the kinetic parameters of *Ta*BG3. Consistent with competitive inhibition, increasing glucose concentration resulted in increased *K*_M(h)_ and *K*_M(t)_, with no or little effect on *k*_cat(h)_ and *k*_cat(t)_; approximate *K*_i_ values of 0.7 mM and 12 mM were found for glucose inhibition of the hydrolytic and transglycosylation reactions, respectively. For further investigation, we used a simplified approach and measured *IC*_50_ values by varying the concentration of glucose in the experiments at a single substrate concentration. Provided that the inhibition is competitive and the substrate concentration is well below its *K*_M_ value, the *IC*_50_ value is close to the true *K*_i_ value [26]. At low substrate concentrations, the contribution of transglycosylation is negligible and is not expected to interfere with glucose inhibition of the hydrolytic reaction. First, the *K*_M(h)_ values for pNPG were measured using a non-linear regression analysis of the data of pNPG hydrolysis, according to Equation 2 (the rate constant of pNPG hydrolysis, *k*^obs^_pNPG_, was plotted as a function of [pNPG] instead of *k*^obs^_CB_*versus* [CB]) (Figure 1A). At 25°C, *K*_M(h)_ values of 0.61 ± 0.06 mM, 0.22 ± 0.03 mM and 0.095 ± 0.003 mM were found for *N188*BG, *Ta*BG3 and *At*BG3, respectively. In the inhibition studies with *N188*BG, 50 μM pNPG was used as the substrate; however, low *K*_M(h)_ values did not permit the use of the pNPG substrate for *Ta*BG3 and *At*BG3 because of the sensitivity limitations of the initial rate measurements under the conditions of [pNPG] < <*K*_M(h)_. As the detection of MU fluorescence enables much greater sensitivity, MUG concentrations of 5 μM and 2.5 μM were used for *Ta*BG3 and *At*BG3, respectively. The initial rates measured in the presence of glucose (*v*_i_) were divided by those measured in the absence of glucose (*v*_0_), and data in the coordinates *v*_i_/*v*_0_*versus* [Glc] (Figure 3) were fitted to Equation 3.

(3) $$\frac{v_{i}}{v_{0}}=\frac{\left[S\right]+C_{1}}{\left[S\right]+C_{1}\left(1+\frac{\left[\mathrm{Glc}\right]}{C_{2}}\right)}$$

In the fitting of the data, the substrate concentration ([S]) was fixed to the value used in the experiments. The value of [S] and the values of the empirical constants *C*_1_ and *C*_2_ found by the fitting were further used to calculate the *IC*_50_ value using Equation 4.

(4) $$IC_{50}=C_{2}\left(1+\frac{\left[S\right]}{C_{1}}\right)$$

**Figure 4:**
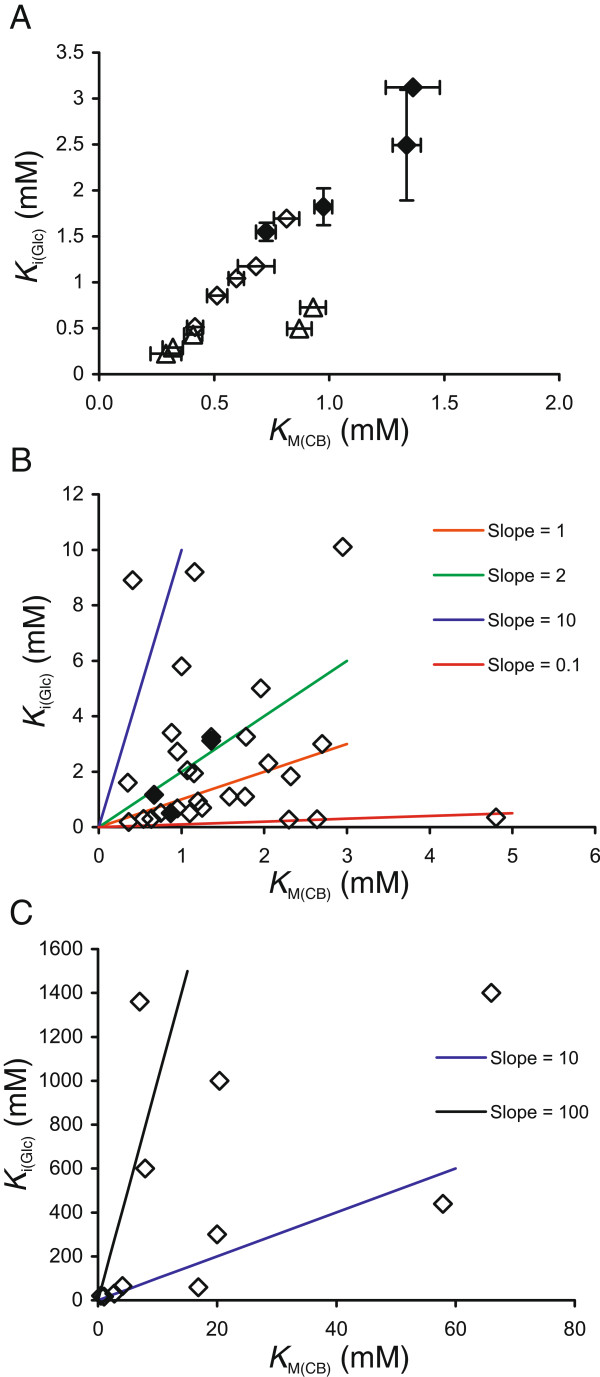
**A higher affinity for cellobiose is accompanied by a stronger glucose inhibition of β-glucosidases (BGs). (A)** The values of the Michaelis constants for cellobiose hydrolysis (*K*_M(h)_) and the inhibition constants for glucose (*K*_i(Glc)_) are from Table 1 and Table 4, respectively. *Ta*BG3 (◊), *At*BG3 (∆) and *N188*BG (♦). **(B** and **C)** A literature survey revealed that BGs can be tentatively divided into three groups based on their relative affinities for cellobiose (*K*_M(CB)_) and glucose (*K*_i(Glc)_): (i) *K*_M(CB)_ > > *K*_i(Glc)_, BGs near the red line; (ii) *K*_M(CB)_ ≈ *K*_i(Glc)_, BGs near the pink and the green line and (iii) *K*_M(CB)_ < <*K*_i(Glc)_, BGs near the blue and the black line. For the numerical values of *K*_M(CB)_ and *K*_i(Glc)_, see Table 5. If *K*_i(Glc)_ values measured using both pNPG and cellobiose as the substrate were available, the priority was given to the *K*_i(Glc)_ value measured using cellobiose. Data from the present study (♦).

Because of the experimental conditions, [S] < <*K*_M_, these *IC*_50_ values are further referred to as *K*_i_ for glucose, *K*_i(Glc)_. The *K*_i(Glc)_ values for BGs at different temperatures are listed in Table 4; the enzyme most sensitive to glucose inhibition was *At*BG3, followed by *Ta*BG3 and *N188*BG. With all BGs, the strength of glucose inhibition decreased with increasing temperature; thus, the use of higher temperatures has an advantage of both increasing the catalytic efficiency and relieving the product inhibition. By plotting *K*_i(Glc)_*versus K*_M(h)_ for cellobiose, *K*_M(CB)_ (Figure 4A) revealed a trade-off between the two parameters: a higher affinity for cellobiose is accompanied by a stronger glucose inhibition. Because of the similar temperature dependency of *K*_M(CB)_ and *K*_i(Glc)_, the data points for a specific BG at different temperatures followed the same line in the coordinates *K*_i(Glc)_*versus K*_M(CB)_ (Figure 4A). We also conducted a literature survey in search of a correlation between the kinetic parameters of cellobiose hydrolysis and glucose inhibition. Table 5 lists BGs in order of increasing *K*_i(Glc)_. Although much scattering is observed, BGs can be tentatively divided into three groups based on their relative affinity for cellobiose (*K*_M(CB)_) and glucose (*K*_i(Glc)_). (1) BGs with a higher affinity for glucose than for cellobiose, *K*_M(CB)_ > > *K*_i(Glc)_ (Figure 4B, BGs near the red line). Because of the low specificity constants for cellobiose and strong glucose inhibition, these BGs are not suitable for supporting CBHs in cellulose degradation. (2) BGs with an approximately equal affinity for cellobiose and glucose, *K*_M(CB)_ ≈ *K*_i(Glc)_. Most of the listed BGs belong to this group, which can be further divided into two sub-groups, BGs with *K*_M(CB)_ slightly higher than *K*_i(Glc)_ (Figure 4B, BGs near the pink line) and BGs with *K*_M(CB)_ slightly lower than *K*_i(Glc)_ (Figure 4B, BGs near the green line). Although the variation is more than two orders of magnitude (partly because of the different temperatures used), BGs belonging to this group have highest specificity constants for cellobiose (*k*_cat_/*K*_M(CB)_ values usually higher than 10^5^ M^-1^ s^-1^). These BGs include *N188*BG and the other fungal BGs most often used to support cellulases in cellulose hydrolysis. (3) BGs with a higher affinity for cellobiose than for glucose, *K*_M(CB)_ < <*K*_i(Glc)_ (Figure 4B and C, BGs near the blue and black line). This group consists of BGs that are also referred to as glucose-tolerant BGs. Their *K*_i(Glc)_ values are in the molar or sub-molar range, and the *K*_i(Glc)_/*K*_M(CB)_ ratio is often more than 10 [27-33]. These BGs, however, tend to have low *k*_cat_ and *k*_cat_/*K*_M(CB)_ values for cellobiose (*k*_cat_/*K*_M(CB)_ in the order of or below 10^4^ M^-1^ s^-1^).

**Table 4:** Glucose inhibition of β-glucosidases

	***K***_**i **_**for glucose, *K***_**i(Glc)**_**(mM)**		
**t (°C)**	***N188*BG**^**a**^	***Ta*BG3**^**b**^	***At*BG3**^**b**^
25	1.55	0.51	0.22
35	1.82	0.85	0.29
45	2.50	1.04	0.43
55	3.12	1.17	0.50
65		1.69	0.73

^a^studied using pNPG.

^b^studied using MUG.

**Table 5:** Kinetic parameters of selected β-glucosidases

**Organism**			***k***_**cat **_**(s**^**-1**^**)**		***K***_**M **_**(mM)**		***k***_**cat**_**/*K***_**M **_**(10**^**5**^ **M**^**-1**^ **s**^**-1**^**)**		***K***_**i **_**glucose (mM)**		***K***_**i**_**/*K***_**M**_^**a**^	**Ref**
	**t°C**	**pH**	**CB**	**pNPG**	**CB**	**pNPG**	**CB**	**pNPG**	**on CB**	**on pNPG**		
*Penicillium verruculosum*			118^b^	650^b^	0.36	1.6	3.29	4.06		0.19	0.53	[34]
*Phanerochaete chrysosporium*	22	4	50	132	2.3	0.10	0.22	13.8		0.27	0.12	[35]
*Myceliophthora thermophila*	40	5	46	147	2.64	0.39	0.17	3.76		0.28	0.11	[36]
*Thermoascus aurantiacus*	60	4.5	284	242	0.64	0.11	4.46	21.2		0.29	0.45	[37]
*Trichoderma reesei*	50	4.5	22		0.54		0.41		0.29		0.54	[38]
*Fomitopsis palustris*	50	5	102	721	4.8	0.12	0.21	61.6		0.35	0.07	[39]
*Acremonium thermophilum*	55	5	666		0.87		7.65			0.50	0.57	T^c^
*Magnaporthe grisea*	50	5			1.1				0.5		0.45	[8]
*Trichoderma reesei*	40	5	42	118	0.75	0.09	0.56	13.1		0.51	0.68	[40]
*Chaetomium globosum*	50	5	168		0.95		1.77		0.68		0.72	[21]
*Trichoderma reesei*	40	5	29	70.8	1.25	0.1	0.23	6.94		0.7	0.56	[41]
*Penicillium verruculosum*	40	5	89	160	1.2	0.44	0.74	3.64		0.93	0.78	[40]
*Aspergillus fumigatus*	50	5	768		1.77		4.34	0.00	1.1		0.62	[21]
*Penicillium brasilianum*	22	4.8	53.7^b^	146^b^	1.58	0.09	0.34	16.2	1.1	2.3	0.70	[20]
*Thermoascus aurantiacus*	55	5	1058		0.67		15.5			1.17	1.75	T^c^
*Aspergillus niger (N188)*	22	4.8			0.35	0.45			1.6	1.1	4.57	[20]
*Emericella nidulans*	50	5	87		2.32		0.38		1.83		0.79	[21]
*Aspergillus niger (N188)*	50	5	558		1.15		4.85		1.94		1.69	[21]
*Fusarium oxysporum*	50	5	323	7.7	1.07	0.09	3.02	0.83		2.05	1.92	[42]
*Penicillium brasilianum*	50	5	520		2.05		2.54		2.3		1.12	[21]
*Aspergillus japonicus*	40	5	350	259	0.95	0.6	3.68	4.32		2.73	2.87	[40]
*Aspergillus niger*	25	4.5	104^b^	61^b^	2.7	1	0.38	0.61		3	1.11	[43]
*Aspergillus niger (N188)*	55	5	691		1.36		5.07			3.12	2.29	T^c^
*Trichoderma reesei*	50	4.8	41	87.9	1.36	0.38	0.30	2.31		3.25	2.39	[23]
*Aspergillus oryzae*	50	5	363		1.78		2.04		3.26		1.83	[21]
*Aspergillus niger (N188)*	50	4.8	32	23.4	0.88	0.57	0.36	0.41	3.4	2.7	3.86	[23]
*Aspergillus oryzae*	50	5	1000	370	1.96	0.29	5.10	12.7	5	2.9	2.55	[22]
*Aspergillus niger*	40	4	2780	917	15.4	2.2	1.81	4.17		5.7	0.37	[44]
*Aspergillus tubingensis*	30	4.6	331^b^	140^b^	1	0.76	3.31	1.83		5.8	5.80	[45]
*Penicillium italicum*	60	4.5	2641	1746	0.41	0.11	64.4	158		8.9	21.7	[25]
*Aspergillus japonicus*	30	5	46^b^	54.5^b^	1.16	0.2	0.40	2.72		9.2	7.93	[46]
*Neurospora crassa*	50	5	423	640	2.95	2.54	1.43	2.52	10.1	6.43	3.42	[21]
*Aspergillus sp*	60	4.5				1.0				17	17	[47]
*Periconia sp*	40	5	972^b^	1180^b^	0.5	0.19	19.4	62.7		20	40.0	[48]
*Baltic sea metagenome*	30	6.5	11.2	22.5	2.76	0.37	0.04	0.61		30	10.8	[49]
*Aspergillus niger (N188)*	45	5			16.8	1.77			59.5	1.59	3.54	[24]
*Streptomyces sp*	50	6.5	35.6	28.4	4.1	0.15	0.09	1.89		65	15.8	[50,51]
*Torulopsis wickerhamii*				362^b^	300	2.8		1.29		190	0.63	[52]
*Thermoascus aurantiacus*	40	5	0.72^b^	5.08^b^		0.2		0.25		300		[27]
*Pyrococcus furiosus*	95	5	454	677^b^	20	0.15	0.23	45.1		300	15.0	[53]
*Debaromyces vanrijiae*	40	5	141^b^	1113^b^	57.9	0.77	0.02	14.5		439	7.58	[54]
*Aspergillus niger*	40	4	4.3^b^	223		21.7		0.10		543		[28]
*Thermoanaer. thermosacch.*	70	6.4	104^b^	55^b^	7.9	0.63	0.13	0.88		600	75.9	[29]
*Uncultured bacterium*	40	6.5	13.2^b^	43^b^	20.4	0.39	0.01	1.11		1000	49.0	[55]
*Aspergillus oryzae*	50	5	253^b^	764^b^	7	0.55	0.36	13.9		1360	194	[30]
*Candida peltata*	50	5	54^b^	158^b^	66	2.3	0.01	0.69		1400	21.2	[31]

BGs are listed in the order of increasing *K*_i(Glc)_. If *K*_i(Glc)_ values measured using both pNPG and cellobiose (CB) as the substrate were available, the priority was given to the *K*_i(Glc)_ value measured using cellobiose.

^a^*K*_M_ is for cellobiose hydrolysis. If *K*_i(Glc)_ values measured using both pNPG and cellobiose (CB) as the substrate were available, the priority was given to the *K*_i(Glc)_ value measured using cellobiose.

^b^Calculated from the reported specific activity and molecular weight of the enzyme.

^c^This study.

**Figure 5:**
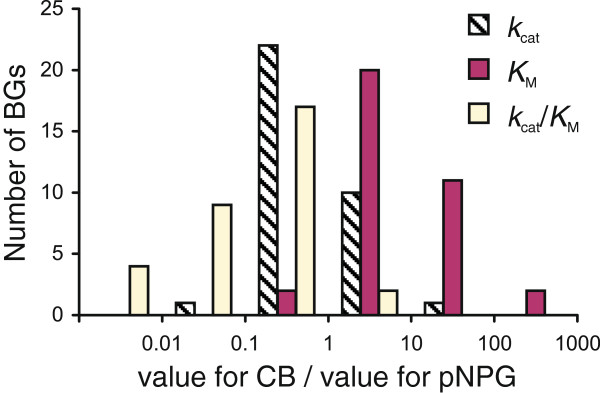
**Comparison of the kinetic parameters of β-glucosidases measured for cellobiose and pNPG.** The value of the parameter measured for cellobiose was divided by the value of the corresponding parameter measured for pNPG. *k*_cat_ denotes *k*_cat(CB)_/*k*_cat(pNPG)_, *K*_M_ denotes *K*_M(CB)_/*K*_M(pNPG)_, and *k*_cat_/*K*_M_ denotes (*k*_cat(CB)_/*K*_M(CB)_)/(*k*_cat(pNPG)_/*K*_M(pNPG)_). The parameter values listed in Table 5 were used for the calculation of the ratios.

BGs have been divided into three groups based on their substrate specificity [9]: (i) aryl BGs, (ii) true cellobiases and (iii) broad-substrate specificity BGs. Although there is no stringent, unequivocal criteria for this classification, the BGs listed in Table 5 appear to belong to the last group. A comparison of the kinetic parameters for cellobiose and pNPG hydrolysis revealed that pNPG is the preferred substrate for the most of the listed BGs (Figure 5). The higher specificity constants for pNPG were mainly caused by the lower *K*_M_ values for pNPG, whereas the *k*_cat_ values for pNPG and cellobiose were of the same order. The preference for pNPG over cellobiose was most prominent in the case of the glucose-tolerant BGs and also for BGs with *K*_M(CB)_ > > *K*_i(Glc)_.

In addition to protein properties, such as stability with regard to pH and temperature, the kinetic properties of enzymes must also be considered in selecting BGs to support cellulases. The main “work horses” in cellulose hydrolysis, GH7 CBHs, are inhibited by cellobiose, with *IC*_50_ values in the few millimolar range [26,56-58], and most BGs have a *K*_M_ value for cellobiose in the same range (Table 5). Thus, to be efficient in relieving the cellobiose inhibition of CBHs, a BG must maintain the steady-state cellobiose concentration well below its *IC*_50_ value for CBHs, meaning that most BGs must operate under the conditions of [CB] < <*K*_M(CB)_. Under the conditions of [CB] < <*K*_M(h)_, and bearing in mind that *K*_M(h)_ < <*K*_M(t)_ and *k*_cat(t)_ < <*k*_cat(h)_, Equation 2 reduces to

(5) $$k_{\mathrm{CB}}^{\mathrm{obs}}\approx \frac{k_{\mathrm{cat}\left(h\right)}}{K_{M\left(h\right)}}\left[\mathrm{CB}\right]$$

**Figure 6:**
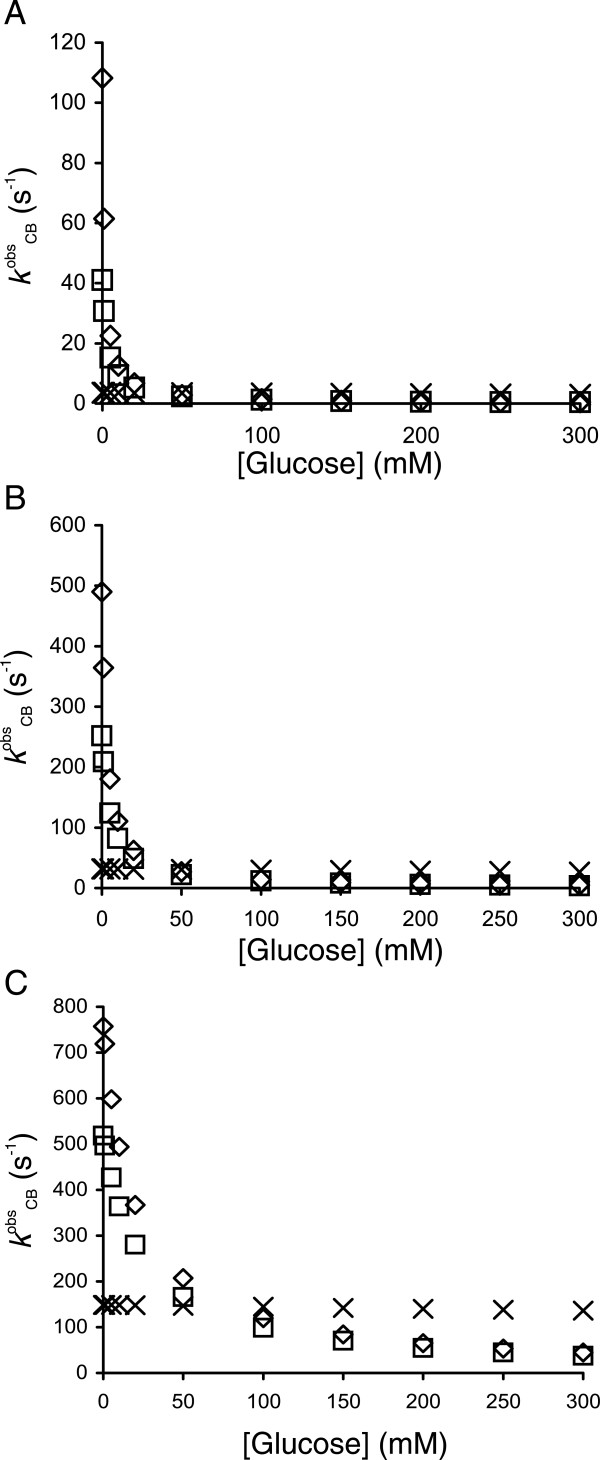
**Calculated values of the rate constants of cellobiose hydrolysis for β-glucosidases with different kinetic properties.** The values of the observed rate constants of cellobiose hydrolysis (*k*^obs^_CB_) at different cellobiose and glucose concentrations were calculated using the simple Michaelis-Menten equation with competitive glucose inhibition and ignoring substrate inhibition. The β-glucosidases used were *Ta*BG3 (◊) and *N188*BG (□), characterized in the present study, and a previously characterized glucose-tolerant β-glucosidase from *Aspergillus oryzae* (*Ao*BG3) (×) [30]. *k*_cat(h)_ values of 806 s^-1^, 587 s^-1^ and 253 s^-1^, *K*_M(CB)_ values of 0.65 mM, 1.33 mM and 7.0 mM and *K*_i(Glc)_ values of 1.14 mM, 2.75 mM and 1360 mM were used for *Ta*BG3, *N188*BG and *Ao*BG3, respectively. The concentration of cellobiose was set to 0.1 mM **(A)**, 1.0 mM **(B)** or 10 mM **(C)**.

Thus, under the conditions of low cellobiose concentrations, the rate of cellobiose hydrolysis is governed by the specificity constant for the hydrolytic reaction, and the terms accounting for transglycosylation cancel out. Therefore, the *k*_cat(h)_/*K*_M(h)_ value may be an important characteristic for selecting BGs to support cellulases in cellulose hydrolysis. Although the glucose inhibition of CBHs is relatively weak [26,58], the glucose inhibition of a BG will eventually lead to the accumulation of cellobiose and CBH inhibition. Therefore, the value of *K*_i(Glc)_ is another important characteristic to consider when selecting BGs. We predicted the *k*^obs^_CB_ values at different cellobiose and glucose concentrations for three BGs with different *k*_cat_, *K*_M(CB)_ and *K*_i(Glc)_ values (Figure 6). Because of the unavailability of the values of the kinetic parameters, the transglycosylation reaction was ignored, and a simple Michaelis-Menten equation with competitive glucose inhibition was used in the calculations. Using a numerical analysis of the time courses of cellobiose hydrolysis, Bohlin et al. found that product inhibition exerts a more pronounced negative effect on BG activity than transglycosylation [8]. Nonetheless, by ignoring transglycosylation, the *k*^obs^_CB_ values calculated herein are somewhat overestimated. *Ta*BG3 and *N188*BG (characterized in this study) and a glucose-tolerant BG from *Aspergillus oryzae* (*Ao*BG3) were assessed [30]. The values of the kinetic parameters for *Ta*BG3 and *N188*BG at 50°C were calculated based on data for the temperature dependency of the parameters. *Ta*BG3 had the highest specificity constant (*k*_cat_/*K*_M(CB)_ = 1.25 x 10^6^ M^-1^ s^-1^) but was the enzyme most sensitive to glucose inhibition (*K*_i(Glc)_ = 1.14 mM). In contrast, *Ao*BG3 was highly tolerant to glucose inhibition (*K*_i(Glc)_ = 1.36 M) but had a moderate specificity constant (*k*_cat_/*K*_M(CB)_ = 3.6 x 10^4^ M^-1^ s^-1^). Amid these two enzymes was *N188*BG, with *k*_cat_/*K*_M(CB)_ and *K*_i(Glc)_ values of 4.4 x 10^5^ M^-1^ s^-1^ and 2.76 mM, respectively. The *k*^obs^_CB_ of *Ta*BG3 was higher than that of *N188*BG under all the conditions tested, but the difference was more prominent at low cellobiose and glucose concentrations. Although *Ao*BG3 had much lower *k*^obs^_CB_ values at low glucose concentrations, it outperformed *Ta*BG3 and *N188*BG at glucose concentrations above 50 mM. Thus, *Ao*BG3 appears to be a better candidate BG for the hydrolysis of cellulose in separate hydrolysis and fermentation processes under high dry matter conditions. The amount of BG required to maintain the cellobiose concentration at a certain steady-state level depends on the velocity of cellobiose production from cellulose. The maximum catalytic potential of CBHs is given by their *k*_cat_ value of cellulose hydrolysis and is within the range of 1–10 s^-1^[57,59,60]. If *k*_cat_ for cellulose hydrolysis equal to 2 s^-1^ and *k*^obs^_CB_ is 100 s^-1^, then a molar ratio of CBH/BG of 50 is required to maintain a steady-state cellobiose concentration, which means that the relative amount of BG in a cellulase system must be approximately 4% (w/w, considering that BGs usually have approximately 2-fold higher molar masses than CBHs). However, if *k*^obs^_CB_ is only 10 s^-1^, as in the case of *Ta*BG3 and *N188*BG at high glucose concentrations or in the case of *Ao*BG3 at low cellobiose concentrations (Figure 6), the relative amount of BG must be 10 times higher. Although the hydrolysis of lignocellulose is much slower than that predicted by the *k*_cat_ value of CBHs, we used the catalytic potential of CBHs to predict the relative amount of BG to ensure that the rate limitation of cellulose hydrolysis via BG activity is excluded. The selection criteria of candidate BGs also depend on the lignocellulose hydrolysis set-up. A high *k*_cat_/*K*_M(CB)_ value always becomes an advantage and is the primary kinetic parameter for selecting BGs. However, in separate hydrolysis and fermentation at a high dry matter concentration, the advantage of having a high *K*_i(Glc)_ value may overbalance the somewhat lower *k*_cat_/*K*_M(CB)_ value. Because of the trade-off between *K*_i(Glc)_ and *K*_M(CB)_, it is, unfortunately, not possible to maximize both *k*_cat_/*K*_M(CB)_ and *K*_i(Glc)_ in parallel.

## Conclusions

The analysis of the kinetic parameters of BGs in the light of the cellobiose inhibition of CBHs suggested that the specificity constant for cellobiose hydrolysis and the inhibition constant for glucose are the most important parameters in selecting BGs to support cellulose hydrolysis. The use of higher temperatures had the advantage of both increasing the catalytic efficiency and relieving the glucose inhibition of BGs. Our data, together with data from a literature survey, revealed a trade-off between the strength of glucose inhibition and the affinity for cellobiose: an increased tolerance to glucose inhibition was accompanied by a decrease in catalytic efficiency (lower specificity constant values). Therefore, the optimal properties of the candidate BG depend on the cellulose hydrolysis set-up. Although a high specificity constant is always an advantage, the priority may be given to a higher tolerance to glucose inhibition when performing separate hydrolysis and fermentation.

## Methods

### Materials

Glucose, MUG, pNPG, Novozyme®188 and BSA were purchased from Sigma-Aldrich. Cellobiose (≥ 99%) was obtained from Fluka. All the chemicals were used as received from the supplier.

### Enzymes

*N188*BG was purified from Novozyme®188, as previously described [61]. Culture filtrates containing *At*BG3 or *Ta*BG3 were kindly provided by Terhi Puranen from Roal Oy (Rajamäki, Finland). BGs were heterologously expressed in a *Trichoderma reesei* (*Tr*) strain that lacks the genes of four major cellulases [15]. *At*BG3 and *Ta*BG3 were purified using gel-filtration chromatography. The buffer of the crude BG preparation was first changed to 50 mM sodium acetate (pH 5) containing 0.15 M NaCl using a Toyopearl HW-40 column. Fractions with high pNPG-ase activity were combined, concentrated with Amicon centrifugal filter devices (5,000 MWCO) and applied to a Sephacryl S-200 column equilibrated with 50 mM sodium acetate (pH 5) containing 0.15 M NaCl. *Ta*BG3 was purified identically but using a Sephacryl S-300 column. The purity of *At*BG3 and *Ta*BG3 was approximately 95%, as determined by SDS-PAGE. The concentration of *At*BG3 and *Ta*BG3 was determined by the bicinchoninic acid method using BSA as a standard and molecular weights of 101 kDa and 81 kDa, respectively [15]. The concentration of *N188*BG was measured by the absorbance at 280 nm using a theoretical ϵ_280_ value of 180,000 M^-1^ cm^-1^. Several BGs from *T. aurantiacus* have been previously characterized [27,37,62-64]. According to the molecular weight, *Ta*BG3 characterized herein is closest to that characterized by Tong et al. [62].

### Hydrolysis of cellobiose by BGs

The experiments were performed in 50 mM sodium acetate buffer (pH 5.0) containing 0.1 g l^-1^ BSA in a total volume of 0.5 ml. The concentration of cellobiose was varied between 0.1 – 50 mM, and glucose formation was followed in the linear region of time curves. The reaction was stopped by the addition of 0.25 ml 1.0 M Tris–HCl (pH 8.5), and the concentration of glucose was measured using the hexokinase/glucose-6-phosphate dehydrogenase method. The concentrations of hexokinase, G6PDH, NADP^+^, ATP and MgCl_2_ in the assay were 1.5 U/ml, 0.75 U/ml, 0.64 mM, 1.26 mM and 13.3 mM, respectively. After completion of the reaction (approximately 15 min), the absorbance at 340 nm was recorded. The zero data points were identical, but 0.25 ml 1.0 M Tris–HCl (pH 8.5) was added prior to BG. Calibration curves were generated using glucose as a standard.

### Activity and glucose inhibition of BGs using pNPG and MUG

For the activity measurements, the initial rates of pNPG (0.01 – 20 mM) hydrolysis were measured in 50 mM sodium acetate buffer (pH 5.0) containing 0.1 g l^-1^ BSA in a total volume of 0.9 ml. The reactions were stopped by the addition of 0.1 ml 1.0 M NH_3_, and the pNP released was quantified by measuring the absorbance at 414 nm. The glucose inhibition of BGs was measured using 0.05 mM pNPG (*N188*BG), 5 μM MUG (*Ta*BG3) or 2.5 μM MUG (*At*BG3) as the substrate. The experiments were performed as above, but the reactions were supplied with glucose (0.1 – 36 mM). The pNP released was quantified by measuring the absorbance at 414 nm, and the MU released was quantified by fluorescence using excitation and emission wavelengths of 360 nm and 450 nm, respectively. All the rates correspond to the initial rates.

## Abbreviations

At: *Acremonium thermophilum*; BG: β-glucosidase; BSA: Bovine serum albumin; CB: Cellobiose; CBH: Cellobiohydrolase; EG: Endoglucanase; GH: Glycoside hydrolase; Glc: Glucose; MU: 4-methylumbelliferone; MUG: 4-methylumbelliferyl-β-glucoside; N188BG: BG purified from Novozyme®188; pNP: Para-nitrophenol; pNPG: Para-nitrophenyl-β-glucoside; SHF: Separate hydrolysis and fermentation; SSF: Simultaneous saccharification and fermentation; Ta: *Thermoascus aurantiacus*; Tr: *Trichoderma reesei.*

## Competing interests

The authors declare that they have no competing interests.

## Authors’ contributions

HT and PV designed and performed the experiments. PV wrote the paper. Both authors read and approved the final manuscript.

## Supplementary Material

Additional file 1 Supplemental material to “Selecting beta-glucosidases to support cellulases in cellulose saccharification”. Click here for file
