# Supplementary material for: Selecting β-glucosidases to support cellulases in cellulose saccharification
Source: Biotechnol Biofuels. 2013 Jul 24;6:105. doi: 10.1186/1754-6834-6-105 (PMC3726394; doi:10.1186/1754-6834-6-105)
Supplement: Additional file 1 — Supplemental material to “Selecting beta-glucosidases to support cellulases in cellulose saccharification”. [file 1754-6834-6-105-S1.doc]

**Supplemental material**

**Selecting β-glucosidases to support cellulases in cellulose saccharification**

Hele Teugjas and Priit Väljamäe*

Institute of Molecular and Cell Biology, University of Tartu, Riia 23b – 202, 51010 Tartu, Estonia.

*Corresponding author: priit.valjamae@ut.ee

**Table S1. The kinetic parameters for cellobiose hydrolysis by β-glucosidases found using different equations and data sets.** The values of kinetic parameters were obtained by a non-linear regression analysis under three different conditions (A – C).

**A** - All the data points were included in the non-linear regression analysis according to Equation 2.

**B** – The data points with the highest cellobiose concentration up to approximately 5 *K*_M(h)_ (the estimate for *K*_M(h)_ was obtained from the analysis of all the data points, see the conditions in A) were included in the non-linear regression analysis according to Equation 2. Due to insufficient data, the values of the parameters of transglycosylation exhibited very high uncertainty and are not shown (n.s.).

**C** – The data points were selected such that the substrate inhibition was not yet revealed in the case of the data point with the highest cellobiose concentration. A non-linear regression analysis was performed according to the simple Michaelis-Menten equation.

$k_{CB}^{obs}=\frac{k_{cat\left(h\right)}\left[CB\right]}{K_{M\left(h\right)}+\left[CB\right]}$ (S1)

In all tables, n is the number of data points included in the non-linear regression analysis. Concentration refers to the highest concentration of cellobiose included in the non-linear regression analysis. *k*_cat(h)_ and *k*_cat(t)_ are in units of s^-1^, *K*_M(h)_ and *K*_M(t)_ are in units of mM, and the specificity constant for cellobiose hydrolysis, *k*_(h)_ = *k*_cat(h)_/*K*_M(h)_, is in units of s^-1^ mM^-1^.

|  | ***N188*BG 25°C** | | | ***N188*BG 35°C** | | |
| --- | --- | --- | --- | --- | --- | --- |
|  | A (n=13) 30 mM | B (n=9)  3.0 mM | C (n=9)  3.0 mM | A (n=16) 40 mM | B (n=10)  5.0 mM | C (n=10)  5.0 mM |
| *k*_cat(h)_ | 121 | 122 | 101 | 271 | 224 | 231 |
| *K*_M(h)_ | 0.73 | 0.72 | 0.54 | 0.97 | 0.85 | 0.75 |
| *k*_(h)_ | 166 | 169 | 187 | 278 | 262 | 307 |
| *k*_cat(t)_ | 36 | n.s. | - | -36 | n.s. | - |
| *K*_M(t)_ | 12 | n.s. | - | 26 | n.s. | - |

|  | ***N188*BG 45°C** | | | ***N188*BG 55°C** | | |
| --- | --- | --- | --- | --- | --- | --- |
|  | A (n=19) 45 mM | B (n=11)  7.5 mM | C (n=11)  7.5 mM | A (n=19) 45 mM | B (n=11)  5.0 mM | C (n=12)  5.0 mM |
| *k*_cat(h)_ | 493 | 453 | 398 | 691 | 734 | 597 |
| *K*_M(h)_ | 1.34 | 1.26 | 0.94 | 1.36 | 1.48 | 1.07 |
| *k*_(h)_ | 369 | 359 | 425 | 507 | 495 | 557 |
| *k*_cat(t)_ | 210 | n.s. | - | n.s | n.s. | - |
| *K*_M(t)_ | 17 | n.s. | - | 106 | n.s. | - |

|  | ***Ta*BG3 25°C** | | | ***Ta*BG3 35°C** | | |
| --- | --- | --- | --- | --- | --- | --- |
|  | A (n=18) 50 mM | B (n=9)  2.0 mM | C (n=8)  1.0 mM | A (n=18) 50 mM | B (n=10)  3.0 mM | C (n=8)  1.0 mM |
| *k*_cat(h)_ | 227 | 231 | 167 | 401 | 421 | 316 |
| *K*_M(h)_ | 0.42 | 0.44 | 0.26 | 0.51 | 0.55 | 0.37 |
| *k*_(h)_ | 543 | 531 | 645 | 781 | 765 | 861 |
| *k*_cat(t)_ | -10.4 | n.s. | - | 14 | n.s. | - |
| *K*_M(t)_ | 3.0 | n.s. | - | 3.5 | n.s. | - |

|  | ***Ta*BG3 45°C** | | | ***Ta*BG3 55°C** | | |
| --- | --- | --- | --- | --- | --- | --- |
|  | A (n=18) 50 mM | B (n=10)  3.0 mM | C (n=9)  2.0 mM | A (n=18) 50 mM | B (n=11)  5.0 mM | C (n=9)  2.0 mM |
| *k*_cat(h)_ | 632 | 601 | 468 | 1058 | 1021 | 846 |
| *K*_M(h)_ | 0.60 | 0.53 | 0.36 | 0.67 | 0.62 | 0.48 |
| *k*_(h)_ | 1060 | 1126 | 1288 | 1550 | 1650 | 1777 |
| *k*_cat(t)_ | 94 | n.s. | - | 14.8 | n.s. | - |
| *K*_M(t)_ | 4.7 | n.s. | - | 6.9 | n.s. | - |

|  | ***Ta*BG3 65°C** | | |
| --- | --- | --- | --- |
|  | A (n=18) 50 mM | B (n=11)  5.0 mM | C (n=10)  3.0 mM |
| *k*_cat(h)_ | 1497 | 1560 | 1228 |
| *K*_M(h)_ | 0.82 | 0.85 | 0.60 |
| *k*_(h)_ | 1840 | 1829 | 2048 |
| *k*_cat(t)_ | 182 | n.s. | - |
| *K*_M(t)_ | 11 | n.s. | - |

|  | ***At*BG3 25°C** | | | ***At*BG3 35°C** | | |
| --- | --- | --- | --- | --- | --- | --- |
|  | A (n=21) 50 mM | B (n=12)  2.0 mM | C (n=10)  0.75 mM | A (n=21) 50 mM | B (n=12)  2.0 mM | C (n=11)  1.0 mM |
| *k*_cat(h)_ | 105 | 121 | 99 | 180 | 206 | 161 |
| *K*_M(h)_ | 0.29 | 0.36 | 0.29 | 0.32 | 0.39 | 0.27 |
| *k*_(h)_ | 361 | 333 | 344 | 562 | 527 | 586 |
| *k*_cat(t)_ | 2.2 | n.s. | - | 19.2 | n.s. | - |
| *K*_M(t)_ | 3.9 | n.s. | - | 5.9 | n.s. | - |

|  | ***At*BG3 45°C** | | | ***At*BG3 55°C** | | |
| --- | --- | --- | --- | --- | --- | --- |
|  | A (n=18) 50 mM | B (n=9)  2.0 mM | C (n=9)  2.0 mM | A (n=18) 50 mM | B (n=11)  5.0 mM | C (n=10)  3.0 mM |
| *k*_cat(h)_ | 326 | 368 | 281 | 666 | 742 | 506 |
| *K*_M(h)_ | 0.41 | 0.49 | 0.32 | 0.87 | 1.09 | 0.56 |
| *k*_(h)_ | 799 | 752 | 888 | 765 | 683 | 902 |
| *k*_cat(t)_ | 86 | n.s. | - | 250 | n.s. | - |
| *K*_M(t)_ | 7.9 | n.s. | - | 6.3 | n.s. | - |

|  | ***At*BG3 65°C** | | |
| --- | --- | --- | --- |
|  | A (n=18) 50 mM | B (n=11)  5.0 mM | C (n=11)  5.0 mM |
| *k*_cat(h)_ | 968 | 919 | 773 |
| *K*_M(h)_ | 0.93 | 0.94 | 0.64 |
| *k*_(h)_ | 1040 | 982 | 1214 |
| *k*_cat(t)_ | 464 | n.s. | - |
| *K*_M(t)_ | 11 | n.s. | - |


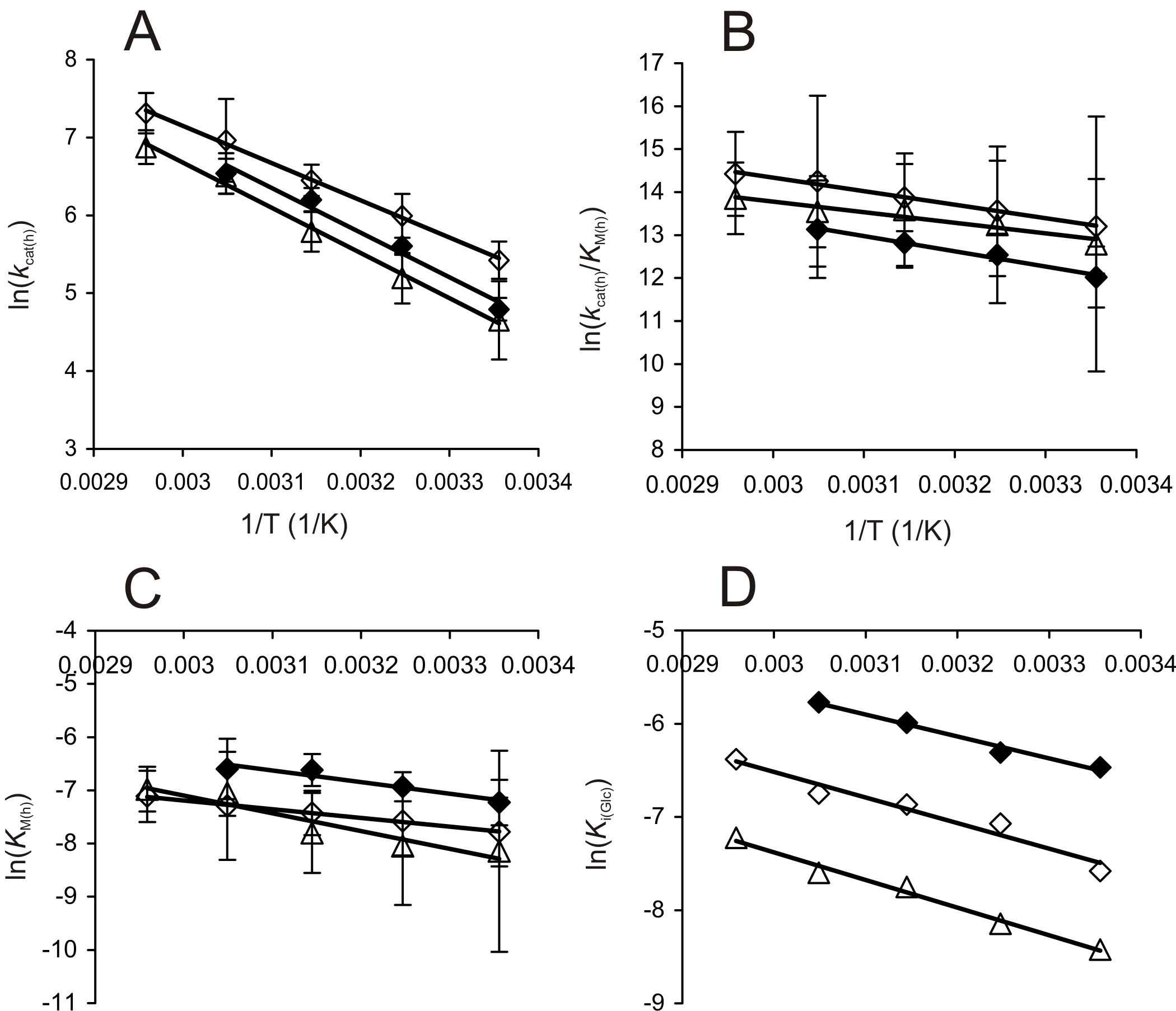


**Figure S1. Arrhenius plots for the kinetic parameters of the β-glucosidase-catalyzed hydrolysis of cellobiose.** The values of the catalytic constants for cellobiose hydrolysis (*k*_cat(h)_, panel A) and Michaelis constants for cellobiose hydrolysis (*K*_M(h)_, panel C) are from Table 1. The values of the specificity constants for cellobiose hydrolysis (*k*_cat(h)_/*K*_M(h)_, panel B) and inhibition constants for glucose (*K*_i(Glc)_, panel D) are from Table 2 and Table 4, respectively. β-glucosidases included *Ta*BG3 (◇), *At*BG3 (△) or *N188*BG (⯁). The solid lines are from the linear regression of the data.

The activation energies, *E*_a_ (kJ mol^-1^), for *k*_cat(h)_ and *k*_cat(h)_/*K*_M(h)_ were obtained from the linear regression according to

$\ln\left(k_{cat\left(h\right)}\right)=C-\frac{E_{a}}{R}\frac{1}{T}$ and $\ln\left(\frac{k_{cat\left(h\right)}}{K_{M\left(h\right)}}\right)=C-\frac{E_{a}}{R}\frac{1}{T}$ , respectively.

The standard binding enthalpies, Δ*H*^0^ (kJ mol^-1^), for *K*_M(h)_ and *K*_i(Glc)_ were obtained from the linear regression according to

$\ln\left(K_{M\left(h\right)}\right)=C-\frac{\Delta H^{0}}{R}\frac{1}{T}$ and $\ln\left(K_{i\left(Glc\right)}\right)=C-\frac{\Delta H^{0}}{R}\frac{1}{T}$ , respectively.

C is an empirical constant, and R is the gas constant.
